# Explaining Individual Differences in Metacognitive Monitoring: A Multilevel Analysis of Person-Level Predictors Across Academic Assessments

**DOI:** 10.3390/jintelligence14080178

**Published:** 2026-08-03

**Authors:** Jinjushang Chen, Qian Zhang, Yue Yin, Jeannine E. Turner, Liang Luo, Chunliang Yang

**Affiliations:** 1 The School of Psychology, Beijing Normal University, Beijing 100088, China; chenashang@outlook.com (J.C.);; 2 The Department of Educational Psychology and Learning Systems, Florida State University, Tallahassee, FL 32306, USA; qzhang4@fsu.edu (Q.Z.);

**Keywords:** metacognitive monitoring, general confidence, resolution, learning-related attributes, beta regression, multilevel modeling, educational assessment

## Abstract

Metacognitive monitoring, students’ evaluations of their own performance, is central to self-regulated learning but often misaligns with actual performance. While situational factors have been studied extensively, less is known about how learning-related beliefs and motivational dispositions—hereafter termed learning-related attributes—relate to individual differences in metacognitive monitoring. This study examined how these learning attributes, alongside cognitive and demographic variables, were associated with students’ general confidence and resolution. Data were drawn from 3946 Chinese sixth-grade students who completed academic assessments in the verbal and mathematical domains, provided post-test performance estimates, and completed questionnaires measuring their learning attributes and demographic characteristics. We conducted multilevel analyses with a random-intercept–random-slope specification and identified several learning attributes as significant predictors of metacognitive monitoring, primarily in terms of general confidence. Adding learning-related attributes after the cognitive and demographic blocks increased marginal R^2^, primarily for general confidence; this order-dependent increment was not interpreted as evidence that the block is inherently more important. The findings extend existing accounts of metacognitive monitoring by suggesting that students’ general confidence is associated with non-cognitive learning attributes—and may thus be partly separable from cognitive ability—whereas their resolution is comparatively less strongly associated with these attributes. Theoretical as well as practical implications are discussed.

## 1. Introduction

People’s metacognitive monitoring—their evaluation of their own cognitive processes—is often inaccurate. Meta-analytic evidence, for example, indicates that students’ self-assessments correlate only weakly to moderately with their actual performance (Dunning et al., 2004). These inaccuracies demonstrate consistency across several domains within educational contexts (e.g., Bellon et al., 2020; Stankov & Crawford, 1996) rather than occurring randomly, suggesting stable patterns of metacognitive bias in learning. Research in cognitive science has revealed that metacognitive biases can arise from both external task features—such as the tendency toward overconfidence on easy tasks and underconfidence on difficult ones—and internal states, such as processing fluency or emotional arousal (Klayman et al., 1999). However, beyond these situational influences, less attention has been paid to person-level characteristics that explain why metacognitive monitoring differs systematically across learners in real-world learning settings.

Research on individual differences in metacognitive monitoring has largely focused on broad dispositions, such as personality traits (e.g., de Bruin et al., 2017), whereas learner characteristics more directly tied to learning processes and outcomes remain underexplored. In this study, we examine self-efficacy, self-esteem, implicit theories of intelligence, motivational orientations, grit, and test anxiety under the descriptive umbrella term learning-related beliefs and motivational dispositions. We use the shorter label learning-related attributes thereafter. This grouping does not imply a unified theory or homogeneous mechanism. Conducting multilevel modeling on a large-scale dataset, we test whether these attributes relate to metacognitive monitoring beyond cognitive and demographic factors and whether their effects differ across two model-derived indicators of monitoring—general confidence and confidence–performance correspondence. By identifying significant learning attributes and distinguishing their effects across different components of metacognitive monitoring, this study addresses a gap in prior research concerning the role of learning-related beliefs and motivations in educational metacognition. 

To address limitations of traditional metacognitive measures in predictive models, we employed multilevel Beta regression with random intercepts and slopes. This approach accommodates the bounded nature of confidence judgments. Confidence judgments were modeled as a function of actual performance on the logit scale, with the random intercept representing individual differences in general confidence and the random slope capturing the correspondence between confidence and performance across assessments. By estimating these components simultaneously, the model provides a joint representation of adjusted baseline estimated performance (general confidence) and within-person confidence–performance correspondence (resolution). This specification intrinsically accounts for task-level measurement error by weighting individual estimates according to their reliability (Murayama et al., 2014), while circumventing the mathematical coupling artifacts inherent in using bias scores as outcomes. This framework enables a more rigorous examination of how person-level characteristics, particularly learning-related attributes, relate to metacognitive monitoring.

### 1.1. Person-Level Metacognitive Monitoring

While metacognitive monitoring is highly sensitive to external task features—such as task difficulty and clarity—which systematically bias confidence judgments (Logg et al., 2018; Moore & Healy, 2008), research also points to substantial inter-individual consistency across domains, suggesting the presence of a person-level, trait-like component (Ais et al., 2016; Mazancieux et al., 2020; Stankov & Crawford, 1996). Consistent with this view, Bellon et al. (2020) found that by third grade (ages 8–9), children showed moderate cross-domain correspondence in metacognitive monitoring between arithmetic and spelling (r = 0.42), as well as a significant predictive relation across domains, whereas younger children showed no such associations. These findings suggest that cross-domain consistency in metacognitive monitoring may begin to emerge during the later years of primary school, providing a rationale for examining whether domain-general person-level factors, particularly learning-related beliefs and motivations, are associated with systematic individual differences in metacognitive judgments across tests in real-world learning contexts.

### 1.2. Operationalization of Person-Level Metacognitive Monitoring

Metacognitive monitoring can be assessed directly through learners’ confidence judgments about the correctness of their responses, or indirectly through indices derived from confidence judgments and task performance that capture distinct dimensions of monitoring accuracy (Nelson, 1990). Absolute accuracy, also termed calibration, reflects the degree of agreement between confidence and actual performance levels. Relative accuracy, also termed resolution or sensitivity, reflects the extent to which confidence discriminates between correct and incorrect responses within an individual. Several operationalizations of monitoring accuracy have predominated in educational research, each with distinct methodological limitations.

Absolute accuracy is commonly assessed using bias scores or calibration curves. A bias score, or difference score, is calculated by subtracting actual performance from estimated performance and indicates both the direction and magnitude of miscalibration. This approach raises a methodological concern in prediction models: when performance variables are used both to compute the bias score and to control for cognitive effects, the resulting statistical dependency between predictors and residuals may produce estimates that reflect statistical artifacts rather than genuine metacognitive relationships (Händel et al., 2020). Calibration curves, on the other hand, depict the relationship between confidence and the proportion of correct responses (e.g., Stone, 2000). Depending on the axis assignment, curves deviating from the diagonal indicate systematic over- or underconfidence. Although visually informative, this technique requires discretizing continuous confidence data into bins. At the intra-individual level, learners rarely use the full range of confidence categories, producing sparse or empty bins (Fleming & Lau, 2014; Masson & Rotello, 2009). Researchers therefore typically pool data at the group level, which obscures the individual differences in calibration that are essential for investigating person-level predictors (Murayama et al., 2014).

Relative accuracy is commonly quantified using indices such as Goodman–Kruskal’s gamma, the area under the receiver operating characteristic curve (AUROC), and meta-d’. Gamma has been widely used in educational research as a nonparametric, within-person measure of the rank-order association between confidence and response accuracy (Fleming & Lau, 2014; Nelson, 1984). Gamma is typically extracted as a single relative-accuracy score per person and entered as a dependent variable in second-stage analyses of individual differences. This treats each person’s gamma as an error-free point estimate and weights all participants equally regardless of how many items contributed to their score (Murayama et al., 2014). More fundamentally, gamma conflates sensitivity with bias such that observed differences in gamma may reflect differences in bias rather than genuine differences in sensitivity (Fleming & Lau, 2014; Masson & Rotello, 2009). 

To address these psychometric limitations, we modeled estimated performance as a function of actual performance across assessment sections using a random-intercept, random-slope multilevel model. By analyzing continuous estimates directly, this approach avoids the discretization required by calibration curves and the mathematical coupling introduced when actual performance is embedded in a bias score. With actual performance person-mean-centered, the random intercept represents an individual’s expected estimate at their mean performance level and thus captures general confidence. The random slope captures section-level resolution—the extent to which estimated performance tracks within-person fluctuations in actual performance. As a multilevel extension of traditional correlational approaches, the model accounts for estimation uncertainty through partial pooling, weighting person-specific estimates by their precision and number of observations (Murayama et al., 2014). Joint estimation distinguishes general confidence from resolution, permits examination of their association, and allows us to test whether they are differentially associated with person-level predictors, particularly learning-related attributes. 

### 1.3. Domain-General Learner Characteristics

Researchers have sought to identify domain-general person-level factors, such as personality traits, that account for individual differences in metacognitive monitoring. However, findings regarding these associations remain inconsistent across domains and contexts. For example, while narcissism was associated with inflated confidence judgment in undergraduate exam scores in one study (de Bruin et al., 2017), this relationship failed to replicate in another sample (Händel et al., 2020). Similarly, optimism has been linked to higher confidence judgment in reasoning-intensive tasks—such as mathematical problem-solving and inference reading questions—regardless of the specific content domain. Yet, this association disappears in tasks heavily reliant on memorization, such as factual reading questions or psychology exams (Golke et al., 2022; Händel et al., 2020). 

Students’ performance is often examined as a key cognitive predictor of their metacognitive monitoring in academic settings. The on-task performance reflects the first-level cognition that provides crucial cues for metacognitive monitoring, and typically shows a positive association with metacognitive monitoring (Nederhand et al., 2021). On the other hand, past academic performance may reflect the prior knowledge that also informs judgments, yet the link between prior knowledge and confidence judgments remains unclear (Witherby et al., 2023), partly because activating accurate prior knowledge is critical yet difficult to observe in real-world learning contexts (van Loon et al., 2013). 

Demographic characteristics especially gender also contribute to variation in academic metacognitive monitoring. For example, gender has been shown to predict systematic differences in confidence judgment, with boys tending to be more overconfident than girls in their school performance (de Bruin et al., 2017; Händel et al., 2020). In the following section, we review studies that examine the role of learning-related attributes in explaining the individual differences in metacognitive monitoring in academic contexts.

### 1.4. Learning-Related Attributes and Metacognition

Although metacognitive judgments are shaped by external task features and internal experiential states, they are also informed by learners’ prior experiences with similar tasks (Fleming & Daw, 2017; van Loon et al., 2013). Thus, individuals may carry relatively stable biases or tendencies into new academic situations. As discussed in the previous section, the consistency observed in person-level metacognitive monitoring may partly reflect such enduring patterns. Although the origins of these individual differences remain unclear, examining relatively stable characteristics that develop within academic contexts may clarify how metacognitive monitoring relates to person-level educational factors. In the following sections, we review several learning-related attributes and consider their potential links to metacognitive monitoring. 

#### 1.4.1. The Role of Self-Referential Beliefs

Prior work on the individual differences in metacognition has documented a close link between self-referential beliefs and metacognitive monitoring (Dapp & Roebers, 2021; Kleitman & Gibson, 2011; Kleitman & Stankov, 2007; Roebers et al., 2012). For example, Kleitman and Stankov (2007) showed that a broad self-confidence factor was predicted not only by task performance and metacognitive awareness, but also by beliefs about one’s reasoning competence. Extending this line of work to school-aged children, Kleitman and Gibson (2011) found that academic self-efficacy and beliefs about one’s memory and reasoning abilities loaded onto a broader metacognitive beliefs factor, which in turn predicted self-confidence. Beyond competence-related beliefs, self-esteem—a more global evaluation of self-worth—has also been linked to performance judgments: Rouault et al. (2022) found that individuals with low self-esteem rated their performance lower than those with high self-esteem despite similar objective performance. Together, these findings suggest that self-referential beliefs may provide an important person-level basis for individual differences in metacognitive monitoring.

This line of research fits naturally within recent hierarchical accounts of metacognition, which distinguish among local confidence judgments, global confidence, and broader self-beliefs. In this framework, Katyal and Fleming (2024) place domain-general self-beliefs at higher levels of the metacognitive hierarchy, above momentary task-level evaluations. This perspective is useful for the present study because it moves beyond viewing metacognitive monitoring as merely a situational response to task features. Instead, it conceptualizes monitoring as embedded within a broader system that includes both local metacognitive experiences and more stable metacognitive knowledge, such as beliefs about one’s own capacities. From this perspective, person-level learning beliefs and motivational orientations may be understood as higher-order self-representations that shape recurring patterns of monitoring across academic assessments. Accordingly, the present study examines whether such person-level factors are associated with systematic between-person differences in metacognitive monitoring across the four assessments, thereby linking task-level performance evaluations to broader learner self-beliefs within a unified metacognitive framework.

#### 1.4.2. The Role of Theories of Intelligence

Implicit theories of intelligence play an important role in self-regulated learning, yet their relation to metacognitive monitoring has received comparatively limited direct attention. These theories distinguish between an entity theory, which views intelligence as fixed, and an incremental theory, which views intelligence as malleable through effort and learning (Dweck, 2006). At a broad level, a meta-analytic review showed that incremental, relative to entity, theories are associated with more adaptive self-regulatory processes, suggesting that mindsets shape how learners respond to challenge and evaluate progress (Burnette et al., 2013). More direct evidence comes from Ehrlinger et al. (2016), who found that individuals with a more fixed view of intelligence showed greater overconfidence than those with a more incremental view. Related recent work further suggests that growth-oriented mindsets can foster more adaptive interpretations of metacognitive disfluency, promoting persistence rather than ability deficit interpretations of difficulty (Jia et al., 2025). Taken together, these findings suggest that theories of intelligence may influence metacognitive monitoring by shaping both learners’ responses to challenge and the informational cues they use to evaluate their own performance.

#### 1.4.3. Motivational Orientations

Similarly, motivational orientations have been examined more often within self-regulation than within metacognition. Ryan and Deci (2000) distinguished intrinsic motivation, in which students are driven by inherent interest, from extrinsic motivation, in which students are driven by external incentives such as grades or recognition. Extending this perspective to metacognition, Miele and Scholer (2018) proposed that students engage in metamotivational monitoring, through which they assess not only how much motivation they have for a task but also what kind of motivation is driving their engagement (e.g., intrinsic vs. extrinsic). In their framework, such monitoring helps learners judge whether their current motivational state fits the demands of the task and, in turn, which motivational orientation or regulation strategy may be most effective. 

Despite its theoretical relevance, direct empirical research linking motivational orientations to metacognitive monitoring remains sparse. Existing evidence suggests, on the one hand, that students’ situational interest may be associated with inflated self-assessments of future performance (Senko et al., 2022), and, on the other hand, that reward structures emphasizing monitoring accuracy rather than performance alone can preserve intrinsic motivation while improving metacognitive monitoring accuracy (Liu et al., 2025). Together, these findings suggest that motivational orientations may shape metacognitive monitoring, although the nature of this relation remains underexplored.

#### 1.4.4. Other Learning Attributes

In addition to self-referential beliefs, theories of intelligence, and motivational orientations, other learning-related beliefs and dispositions may also relate to students’ metacognitive monitoring, albeit more indirectly. Some of these constructs have not been directly linked to metacognitive monitoring, but they may exert influence through their associations with cognitive performance, metacognitive strategy use, or broader self-beliefs such as self-efficacy and self-esteem. The present study therefore adopts a broader exploratory approach by considering a range of learning-related beliefs and orientations that may be relevant to metacognitive judgment while accounting for their interrelations. 

For example, grit, defined as a trait-like characteristic combining perseverance and passion for long-term goals (Duckworth et al., 2007), has been associated with the use of metacognitive strategies such as planning and monitoring (Wolters & Hussain, 2015). Similarly, there is limited research on the relation between test anxiety, a tendency toward excessive worry in evaluative settings (Spielberger & Vagg, 1995), and metacognitive judgment in academic assessments. However, research has shown that state anxiety during text comprehension is associated with lower prediction and postdiction accuracy of task performance (Prinz-Weiß et al., 2023). These findings suggest that broader learning-related beliefs and orientations may also play a role in metacognitive monitoring and therefore warrant further investigation.

## 2. The Present Study

The primary aim of this study was to examine how person-level characteristics—learning-related attributes (e.g., self-efficacy, implicit theories of intelligence), cognitive factors (e.g., performance, prior knowledge), and demographic variables (e.g., gender, SES)—are associated with individual differences in students’ metacognitive monitoring across academic assessments. Cognitive variables were included given that metacognitive monitoring is grounded in learners’ current and prior learning experiences, whereas demographic factors represent distal, stable influences that have been widely examined in educational research. Learning-related attributes were incorporated as a novel focus, as their role in metacognitive monitoring has been insufficiently explored in educational contexts. We selected factors pertaining to beliefs and motivations fostered in educational settings that show relative stability across time as our learning-related attribute variables. 

We analyzed a large-scale dataset from a broader longitudinal project conducted in northern China, drawing on a sample of school-aged students with a mean age of 10 years. Person-level factors were incorporated to predict two model-derived indicators of metacognitive monitoring— general confidence and resolution—modeled as latent parameters within a random-intercept–random-slope multilevel framework. Analyses focused on the Beta regression model, as bounded confidence ratings have been shown to follow a Beta rather than a normal distribution (Fleming & Lau, 2014; Galvin et al., 2003). Full details of the analytic strategy are provided in the Data Analysis Section under the Method Section. This framework enabled us to address the following research questions and corresponding hypotheses.. H1 and H2 were theory-driven, whereas H3 and H4 were prespecified omnibus expectations that the two monitoring indicators would show different patterns of association; several predictors were examined exploratorily.

RQ1: Which learning-related attributes are significant predictors of metacognitive monitoring, after accounting for cognitive, demographic, and other learning attribute covariates?

**H1.**  *Among the learning-related attributes, self-referential beliefs will be the strongest predictors of metacognitive monitoring, given their direct relevance to competence appraisal, whereas implicit theories of intelligence, motivational orientations, and other learning attributes will show comparatively weaker associations. Because prior evidence directly linking these variables to monitoring is limited, the relative contributions of the latter predictors is examined on an exploratory basis.*

RQ2: How much incremental explanatory value do learning-related attributes add when entered after cognitive and demographic factors?

**H2.**  *Learning-related attributes will add explanatory value after cognitive and demographic factors, particularly for general confidence.*

RQ3: Do person-level predictors (learning-related attributes, cognitive, and demographic factors) relate similarly to general confidence and resolution?

**H3.**  *Person-level predictors will show different patterns of association with general confidence and resolution.*

RQ4: Do person-level predictors explain similar proportions of variance in general confidence and resolution?

**H4.**  *Person-level predictors will explain different proportions of variance in general confidence and resolution.*

## 3. Method

### 3.1. Participants

We used data from the Child Academic and Psychological Development Study (CAPDS), a longitudinal project launched in 2016 to track students’ academic and psychological development from childhood through adolescence. The original study recruited 4015 fourth graders from public primary schools in North China using cluster sampling, with data collected from students, parents, teachers, and principals. For this study, we analyzed the sixth wave of data, collected in May 2019 when students were in sixth grade (*n* = 3951). After excluding two cases with missing school information and three cases outside the acceptable age range, the final sample comprised 3946 students (48% girls; M age = 12 years, range = 10–14 years). These students were distributed across 97 classes and 37 school identifiers in the Wave-6 clustering variable. Thus, although the cohort was recruited from 36 schools at baseline, the models used the contemporaneous Wave-6 clustering structure.

### 3.2. Data Collection

Written parental consent was obtained prior to participation. At each wave, trained research assistants administered the study during regular classes in two phases: first, students completed two sets of assessments in Verbal and Mathematics and estimate their overall performance across the four sections of assessments; second, students completed a questionnaire covering key predictor variables (e.g., self-efficacy, self-esteem, motivational orientations) and demographic information.

### 3.3. Instruments

Below, we describe the questionnaire instruments used to address the research purpose. For item-level information, please refer to Supplementary S1.

Performance of academic assessments. Students’ performance was assessed using accuracy scores in the verbal and mathematics domains. The verbal test comprised two sections of multiple-choice items, including 32 knowledge items and 20 reading comprehension items in each section. The mathematics test also included two sections: 21 multiple-choice puzzle items and 7 word problems. Performance was calculated as the percentage of correct responses for the three multiple-choice sections and as the proportion of points earned out of 16 for the word problem section. The accuracy scores across assessments showed good internal consistency (*α* = 0.84).

Metacognitive monitoring. Students provided post-test performance estimates for each section of the assessment. For the first three, they reported the number of correct answers; for word problems, they estimated points earned out of 16. These self-estimates were also transformed into percentage scores. 

Prior knowledge. Prior knowledge was measured using standardized Verbal and Mathematics tests administered at the end of the first semester of Grade 6. The two-item construct demonstrated acceptable reliability (*α* = 0.75). Raw scores (out of 120) were rescaled to 100, then averaged across subjects to yield a composite prior knowledge score.

Academic self-efficacy. We used an adapted subscale from the Self-Efficacy Questionnaire for Children (Muris, 2001). We adapted the Chinese version of the Academic Self-Efficacy subscale into a four-point Likert scale to capture endorsement tendencies. The original seven items, rated on a five-point scale, assess students’ perceived efficacy in tasks such as passing tests, learning subjects, and completing homework (e.g., “How well do you succeed in understanding all subjects in school?”). The scale showed good reliability (*α* = 0.87), and the mean score was used in analysis.

Self-esteem. We used the Chinese version of the Self-Esteem Scale (Shen & Cai, 2008) adapted from Rosenberg (1979). The 10 items, rated on a 4-point Likert scale, measure positive and negative self-image (e.g., “On the whole, I am satisfied with myself”). Although originally treated as unidimensional, the Chinese version fits a bi-dimensional model (Yang & Wang, 2007). For this study, we focused on the positive self-image subscale (5 items; *α* = 0.87), using the sum score in analyses.

Motivational orientation. Students’ motivational orientation was assessed with the scale by Guay et al. (2010). To capture general academic motivation, Verbal and Mathematics subscales were combined into three subscales—extrinsic motivation, identified regulation, and intrinsic motivation—each with six items rated on a five-point Likert scale (e.g., “Reading/Math interests me a lot”). Confirmatory factor analysis suggested reasonably fit for the three-factor structure (*χ*^2^(130) = 3718.61, *p <* 0.001; CFI = 0.943; TLI = 0.933; RMSEA = 0.087; SRMR = 0.083), with loadings of 0.63–0.80. Reliability was acceptable (*α* = 0.85–0.88), and subscale mean scores were used in analyses.

Implicit theories of intelligence. We used the Chinese version of the Mindset Scale (Chen & Wong, 2015), adapted from Dweck’s (2013) scale. The scale includes two four-item subscales measuring growth and fixed mindsets, rated on a five-point Likert scale from strongly disagree (1) to strongly agree (5). A sample item is “People can change how smart they are.” Reliability was acceptable (*α* = 0.81 for growth mindset; *α* = 0.76 for fixed mindset). 

Grit. We measured grit using the Chinese version of the 12-item Grit Scale (Duckworth et al., 2007). Students rated perseverance of effort (e.g., “Setbacks don’t discourage me”) and consistency of interest (reverse-coded; e.g., “My interests change from year to year”) on a four-point scale (1 = disagree to 4 = agree). Reliability was high for both subscales (*α* = 0.89 for perseverance; *α* = 0.87 for consistency).

Test anxiety. We select the subscale of test anxiety from the original scale of Motivated Strategies for Learning Questionnaire (MSLQ) by Pintrich (2000). Students rated items on a four-point scale (1 = disagree to 4 = agree; e.g., “I worry a great deal about tests”). Reliability was high (*α* = 0.87).

Demographic variables. Gender was dichotomously coded, with girls as the reference group. Parental educational level was recoded by summing the values of the original variable reported by each parent, whereby the original scale included seven levels: “no education,” “primary school,” “secondary school,” “high school or vocational education,” “two-year undergraduate degree,” “four-year undergraduate degree,” and “postgraduate degree.” Family annual income was reported in ten ordinal categories. Parental education, family income, and age were treated as continuous variables in the analysis.

### 3.4. Data Analysis

We estimated two multilevel models to examine person-level predictors of metacognitive judgment across assessments with a focus on the beta regression model. Analyses were conducted in RStudio (R 4.3.2) using lme4 for the linear confidence model and glmmTMB for the beta confidence model of bounded performance estimates. Blocks of variables were sequentially added at the person level (cognitive, then demographic, then learning-related attribute factors). For both models, we decomposed the performance variable into between-person (BetweenPersonP_ti_) and within-person (WithinPersonP_ti_) components to distinguish intra-individual from inter-individual effects of performance (Hofmann & Gavin, 1998), and to control for performance effects more accurately when evaluating the influence of other predictors in individual level. Continuous predictors were standardized for comparability. Between-class and between-school clustering effects were also accounted for. Analyses were performed on each imputed dataset and pooled with Rubin’s rules. The complete equations and pooled final-model variance components are provided in Supplementary S2. To complement the order-dependent sequential PRVs, we calculated marginal R^2^ (variance attributable to fixed effects) and conditional R^2^ (variance attributable jointly to fixed and random effects) for each model in the nested sequence.

#### 3.4.1. Linear Confidence Model

To properly estimate the person-level effects while accounting for performance without introducing the artificial statistical effect, we adopted a multilevel modeling approach in which confidence scores were regressed on task-level performance at Level 1 (Equation (1)). At Level 2, the model estimated for each individual two latent parameters: a random intercept and a random slope (Equations (3) and (4)). The resulting random intercept (*β*_0i_) represents each individual’s adjusted baseline level of estimated performance (general confidence). The random slope (*β*_1i_) quantifies within-person confidence–performance correspondence (resolution). By regressing estimated performance on actual performance at Level 1, the resulting random slope operationalizes section-level confidence–performance correspondence. Thus, the multilevel model separates a student’s general confidence (random intercept), from resolution (random slope).

Cognitive factors (BetweenPersonP_ti_ + PriorKnowledge_i_), demographic variables (Demographics_pi_) and learning-related attributes (LearningAttributes_qi_) were then included as predictors of these two latent outcomes. Subscripts *t* and *i* are used to index assessments and individuals, respectively, while subscripts *p* and *q* differentiate demographic and learning attribute variables. As noted earlier, the assumption of normally distributed errors for this linear confidence model may be problematic, as confidence ratings are bounded and skewed. Preliminary diagnostics further indicated heteroskedasticity of residuals (see Figure S1A in Supplementary S3). These issues motivated the refinement to a beta confidence model, described in the following paragraph.

Each student contributed four Level 1 observations, one per assessment section. With this cluster size, classical random-coefficient reliability for person-specific slopes is strongly constrained by the limited within-person sum of squares and would primarily reflect design-induced shrinkage. We therefore did not treat the empirical Bayes slopes as error-free individual scores or use their reliability to claim trait stability. Instead, we evaluated whether allowing slope heterogeneity improved the population model by comparing random-intercept-only and random-intercept–random-slope baseline models with a likelihood-ratio test.

#### 3.4.2. Beta Confidence Model

We then reanalyzed estimated performance proportions using a beta mixed-effects model with a logit link (Equations (2)–(4)). Beta regression was chosen because it keeps predicted values between 0 and 1 and accommodates the mean-dependent variability typical of bounded judgments (Smithson & Verkuilen, 2006). Treating the proportions as discretized observations of an underlying bounded continuum provided a common scale across sections. In the Beta distribution, each response *Y* is assumed to follow *Y*~Beta (*μ*, *ϕ*), where the conditional variance depends jointly on the mean parameter (*μ*) and the dispersion parameter (*ϕ*):

Var(Y|μ, φ) = μ(1 − μ)/(1 + φ).

We allowed the dispersion parameter (*ϕ*) to vary across individuals, using maximum likelihood estimation to model non-constant error variance and thereby mitigate the inflation of standard errors often observed in linear models (Smithson & Verkuilen, 2006). This adjustment was supported by diagnostic evidence showing reduced heteroskedasticity in the residuals (see Figure S1B,C in Supplementary S3). All beta confidence models converged successfully across the 20 imputed datasets with positive-definite Hessians. The pooled reference precision was 11.82, and the student-level standard deviation in log precision was 0.945. Because this component served only to account for unexplained differences in response variability, person-specific precision estimates were neither reported nor interpreted.

Because standard beta regression excludes exact 0 and 1 values, endpoint responses were moved slightly inside this interval in the analysis. Across the completed datasets, 0 and 1 responses represented an average of 0.675% and 10.339% of observations, respectively. We transformed judgment percentage Judgment_ti_ as Y_ti_ = min{0.999, max[0.001, (Judgment_ti_ − 1)/99]}. The prespecified adjustment of 0.001 was negligible relative to the smallest possible increment between section scores (3.125 percentage points) and was applied consistently across all sections and imputed datasets.

Level 1 (Within-Person Level)

*Linear confidence model:*

(1) $${Judgment}_{ti}=\beta_{0i}+\beta_{1i}\cdot {WithinPersonP}_{ti}+\varepsilon_{ti}$$

*Beta confidence model:*

(2) $$Logit(\mu )=\beta_{0i}+\beta_{1i}\cdot {WithinPersonP}_{ti}$$

Level 2 (Between-Person Level)

*Linear confidence & beta confidence model:*

(3) $$\beta_{0i}=\gamma_{00}+\gamma_{01}\cdot {BetweenPersonP}_{i}+\gamma_{02}\cdot {PriorKnowledge}_{i}+\sum_{p}^{P}\gamma_{0p}^{D}\cdot {Demographics}_{pi}+\sum_{q}^{Q}\gamma_{0q}^{T}\cdot {LearningAttributes}_{qi}+u_{0i}$$

(4) $$\beta_{1i}=\gamma_{10}+\gamma_{11}\cdot {BetweenPersonP}_{i}+\gamma_{12}\cdot {PriorKnowledge}_{i}+\sum_{p}^{P}\gamma_{1p}^{D}\cdot {Demographics}_{pi}+\sum_{q}^{Q}\gamma_{1q}^{T}\cdot {LearningAttributes}_{qi}+u_{1i}$$

#### 3.4.3. Missing-Data Management

Missing data were handled using multiple imputation by chained equations (MICE, R 4.3.2). Two score-level validity criteria were applied prior to imputation: (1) composite Likert scores were set to missing if any item was invalid or unanswered, and performance scores were set to missing if >20% of items were invalid or unanswered (isolated omissions coded as 0); (2) in addition to Criterion 1, Likert scales with zero variance were coded as missing to improve validity. These criteria operated on individual variables or composite scores rather than excluding pupils. Therefore, all 3946 pupils with valid clustering information were retained in both multiple-imputation analyses.

Table S1 in Supplementary S4 reports the percentage missing for every analysis variable under Criteria 1 and 2; because all retained missing values were imputed, these are also the variable-specific imputation percentages. Across the 22 variables displayed in Table S1, 3.46% of cells were imputed under Criterion 1 and 5.59% under Criterion 2. Multiple imputation under both Criterion 1 and Criterion 2 showed stable convergence. Sensitivity analyses comparing Criterion 1 (baseline) and Criterion 2 (stricter) produced consistent results (see Tables S2–S4 in Supplementary S4). 

Twenty datasets (20 iterations per chain) were imputed using predictive mean matching. The number of imputations was selected to exceed the largest variable-specific missingness rate (17.83%), thereby limiting Monte Carlo error while retaining computational feasibility. Longitudinal structure guided imputation, with Semester-2 variables imputed forward from Semester-1 measures and vice versa, including all other semester measures and demographic covariates (gender, parental education, family income). The missing-at-random assumption cannot be verified empirically, but it was considered plausible, conditional on the rich observed information in the imputation model: earlier- and later-semester counterparts, other measures from the same semester, and demographic covariates were included because they could be related to both missingness and the incomplete values.

## 4. Results

### 4.1. Descriptive Analysis

All descriptive statistics and correlations reported below are based on pooled results across imputed datasets. Table 1 presents descriptive statistics for the task-level variables. On average, students demonstrated consistent overestimation of their performance across all four sections of academic assessments. The performance across the four assessment sections correlated with one another, ranging from a medium level of 0.53 to 0.70. Similarly, the metacognitive monitoring on the performance across the four assessment sections correlated with one another, ranging from 0.51 to 0.75. The correlation between students’ metacognitive monitoring and their actual performance was relatively small across assessments, ranging from 0.15 to 0.23. 

Table 2 presents the descriptive statistics and correlations among the personal theory variables. Most predictors showed significant and moderate correlations (r = −0.05 to 0.70). However, identified motivation and extrinsic motivation were highly correlated (r = 0.79), suggesting potential theoretical and empirical overlap. When both predictors were included simultaneously, the VIF values approached the collinearity threshold recommended for complex models (identified motivation: VIF = 3.00; extrinsic motivation: VIF = 3.26; threshold = 3.3; Kock, 2015), indicating a potential risk of multicollinearity that could distort parameter estimates. Identified motivation was therefore excluded from subsequent analyses. This decision was also theoretically justified: whereas extrinsic motivation reflects a broader orientation toward externally regulated learning incentives, identified motivation represents a more internalized form of external regulation that partially overlaps with both extrinsic and introjected/intrinsic motivation in self-determination theory, making it the less conceptually distinct variable in the present analytical context. 

### 4.2. Model Comparison

Because individuals may differ in both general confidence (random intercept) and resolution (random slope), we specified the baseline models with random intercepts and random slopes. Before fitting this model, we tested whether random-slope heterogeneity was warranted in the linear and beta confidence models. As shown in Table 3, the random-intercept–random-slope models provided a significantly better fit than the corresponding random-intercept-only models for both the linear confidence model, Δ*χ*^2^(3) = 1301.96, *p* < .001, and the beta confidence model, Δ*χ*^2^(3) = 911.26, *p* < .001. The estimated random-slope variances were τ_11_ = 0.001 and 0.002. These results indicate systematic between-person variation in confidence–performance correspondence across the four assessment sections and support retaining the random slope. However, they do not establish that individual slope estimates are precise or that the slopes reflect a temporally stable or domain-general trait.

Table 3 also shows that, relative to the baseline model, adding person-level cognitive factors significantly improved model fit for both the linear confidence model, Δ*χ*^2^(4) = 183.46, *p* < .001, AIC = 175.46; ΔBIC = 144.79, and beta confidence model, Δ*χ*^2^(4) = 128.12, *p* < .001, ΔAIC = 120.12, ΔBIC = 89.46. Adding Level 2 demographic factors produced further improvements in fit for the linear confidence model, Δ*χ*^2^(8) = 208.8, *p* < .001, and the beta confidence model, Δ*χ*^2^(8) = 291.48, *p <* .001. Finally, the addition of learning-related attributes yielded another significant improvement in fit for both the linear confidence model, Δ*χ*^2^(22) = 431.46, *p <* .001, and the beta confidence model, Δ*χ*^2^(22) = 446.46, *p <* .001. All reported fit indices were based on pooled statistics. 

### 4.3. Model Results

While we report results from both multilevel models, we focus on the beta confidence model, as it provides the most accurate representation of the relationship between person-level predictors and metacognitive judgment. The linear and beta specifications yielded the same central substantive pattern; the beta model chiefly improved distributional fit, bounded prediction, and accommodation of mean-dependent variance. The fixed-effect results address RQ1 and RQ3 by identifying which specific learning-related attributes are significantly associated with metacognitive monitoring (RQ1) and whether the pattern of association for the person-level predictors differs between the two model-derived monitoring indicators—general confidence and resolution (RQ3). The variance component and R^2^ results address RQ2 and RQ4 by estimating the incremental explanatory value of the prespecified predictor sequence (RQ2) and comparing general confidence with resolution where PRV was interpretable (RQ4). Given the large sample size, we consider p value above 0.01 but below 0.05 as marginally significant. All parameter estimates are pooled across imputations.

#### 4.3.1. Fixed Effects on General Confidence

Grand intercept. Table 4 shows that in the beta confidence model, the fixed intercept (logit *β* = 0.91, *S.E.* = 0.04) corresponds to a predicted mean confidence of 0.71 after transformation to the (0,1) scale. This indicates that, on average, students estimated their performance at 71% accuracy across assessments when all predictors were held at their mean or reference level (i.e., the average values of person-level variables for girls). These estimates suggest a general overconfidence: students’ reported confidence systematically exceeded their actual performance (50%) when cognitive, demographic, and learning-related attribute factors were set to their means.

Person-level cognitive factor. In the beta confidence model, controlling for the other predictors, one-standard-deviation (SD) increase in between-person performance was associated with a 0.02 increase in predicted mean confidence, based on the inverse-logit transformation of the fixed intercept and the performance coefficient (logit *β* = 0.10, *S.E.* = 0.02). This indicates that higher performers reported slightly higher confidence on average. By contrast, prior knowledge did not significantly relate to confidence judgments after controlling for actual performance, as reflected in the non-significant coefficient.

Demographics. In the beta confidence model, boys’ general confidence exceeded that of girls by 0.08 (logit *β* = 0.42, *S.E.* = 0.03), holding all other variables constant. By contrast, age, parental education, and family income did not exert significant effects once other person-level predictors were controlled, indicating limited influence of these demographic factors on students’ average confidence judgments.

Learning-related attributes. A one-standard-deviation increase in self-efficacy was associated with a 0.02 increase in mean confidence (logit *β* = 0.11, *S.E.* = 0.02). A similar effect was observed for self-esteem (logit *β* = 0.11, *S.E.* = 0.02). While intrinsic motivation was associated with a modest increase of 0.01 in mean confidence (logit *β* = 0.05, *S.E.* = 0.02), extrinsic motivation showed a stronger association, corresponding to a 0.02 increase (logit *β* = 0.12, *S.E.* = 0.02). Fixed mindset was marginally associated with a 0.01 increase in confidence (logit *β* = 0.04, *S.E.* = 0.02), whereas growth mindset was not a significant predictor. By contrast, test anxiety emerged as a marginally significant negative predictor (logit *β* = –0.04, *S.E.* = 0.02).

#### 4.3.2. Fixed Effects on Resolution

The coefficient for within-person performance (WPP) served as the resolution index (Table 5): when students performed one standard deviation above their own average on a given assessment, their confidence judgments increased by 0.04 (logit *β* = 0.17, *S.E.* = 0.01). Gender emerged as the strongest predictor of confidence–performance correspondence, with boys showing weaker correspondence than girls by 0.01 (logit *β* = –0.03, *S.E.* = 0.01). Among the marginal predictors, higher prior knowledge was associated with slightly stronger resolution (logit *β* = 0.02, *S.E.* = 0.01), whereas a stronger fixed mindset was linked to weaker resolution (logit *β* = –0.01, *S.E.* = 0.01).

#### 4.3.3. Random Effects

The baseline linear confidence model (without predictors) indicated that 53% of the outcome variance was attributable to between-person differences and 42% to within-person variation. For the beta confidence model, the between- and within-person variance proportions are not directly comparable, as Beta regression calculates residual variance differently from standard models. We therefore focus on reporting the between-person variance for the Beta model.

**Figure 1:**
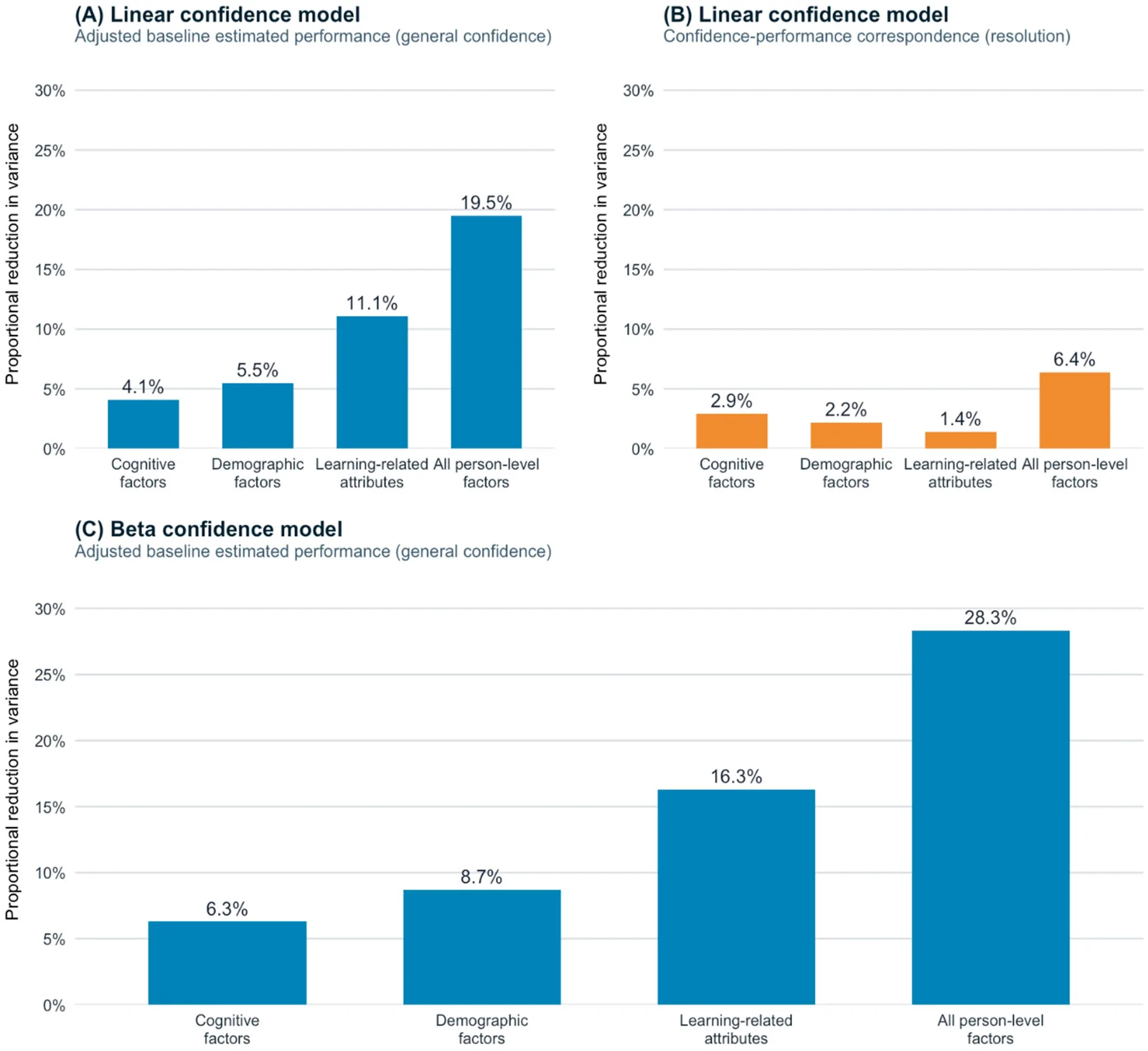
Variance explained by person-level factors by blocks. Note: Block-specific PRVs compare each model with the immediately preceding model. The all-person-level-factors value compares the final model with the baseline. Beta model resolution PRVs are omitted because some block additions increased the estimated random-slope variance, producing negative PRV. Because beta model mean and precision parameters are estimated jointly, such negative values indicate redistribution of latent-scale variance rather than negative explained variance and are not substantively interpreted.

Figure 1 shows the proportion of random variance explained by each category of person-level factors in the linear confidence and beta confidence models. Proportional reduction in variance (PRV) was calculated by comparing each model that included a new category of predictors with the preceding model that excluded it. Overall, person-level predictors collectively explained more variance in general confidence than in resolution. The contrast was 19.5% versus 6.4% in the linear confidence model. 

For the linear confidence model, the cognitive, demographic, and learning-related attribute blocks sequentially reduced general-confidence variance by 4.1%, 5.5%, and 11.1% and resolution variance by 2.9%, 2.2%, and 1.4%. For beta model general confidence, the corresponding sequential reductions were 6.3%, 8.7%, and 16.3%, with all person-level factors jointly reducing random-intercept variance by 28.3% relative to baseline. We do not report resolution PRVs for the beta models because some predictor blocks increased the jointly estimated random-slope variance, yielding negative values that reflect variance redistribution rather than negative explained variance. We therefore emphasize model fit comparisons and $R^{2}$.

As shown in Table 3, sequentially adding the cognitive, demographic, and learning-related attribute blocks significantly improved model fit. Marginal $R^{2}$, the proportion of variance accounted for by fixed effects, increased from 0.02 in the baseline linear model to 0.05, 0.08, and 0.15 across the three successive models. The corresponding values for the beta models were 0.03, 0.06, 0.13, and 0.24. Conditional $R^{2}$, which reflects the combined contribution of fixed and random effects, remained nearly constant at approximately 0.65 for the linear models and 0.97 for the beta models. In the final models, the differences between conditional and marginal $R^{2}$ were 0.50 and 0.73, respectively, indicating the additional contribution of the random-effects structure.

Finally, the correlation between the random intercept (general confidence) and the random slope (resolution) was moderately negative in both models (linear confidence model: r = −0.26; beta confidence model: r = −0.21), indicating that students with lower general confidence tended to show stronger resolution after accounting for all person-level predictors.

As complementary measures of the explanatory contributions of the fixed and random effects, we also estimated marginal and conditional R^2^, respectively. Marginal R^2^ increased from 0.02 in the baseline linear confidence model to 0.05, 0.08, and 0.15 after adding the cognitive, demographic, and learning-related attribute blocks, respectively. In the beta confidence model, marginal R^2^ increased from 0.03 to 0.06, 0.13, and 0.24. Conditional R^2^ remained approximately 0.65 throughout the linear sequence and 0.97 throughout the beta sequence. The increase in marginal R^2^ indicates growing fixed-effect explanatory value, whereas the near-constant conditional R^2^ reflects the dominant and stable contribution of the common random-effects structure.

## 5. Discussion

A central aim of this study was to examine whether person-level characteristics help explain why metacognitive monitoring differs systematically across learners beyond situational influences. The multilevel analyses indicate that learning-related attributes collectively explained substantial between-person variation in metacognitive monitoring, particularly in general confidence. Their contribution exceeded that of cognitive and demographic factors for general confidence but was considerably smaller for resolution. This divergence is theoretically informative, suggesting that learning-related beliefs and motivational orientations are more closely associated with students’ baseline confidence level than with their degree of confidence–performance correspondence across assessments.

Fixed-effects analyses further identified which specific person-level factors were significant correlates of each monitoring indicator. Among demographic predictors, gender emerged as the clearest and most consistent correlate: boys reported higher general confidence but showed weaker resolution than girls, a pattern consistent with well-documented gender differences in academic self-assessment (Händel et al., 2020; Pallier, 2003). The specific contributions of individual learning-related attributes to metacognitive monitoring are discussed in the section below.

### 5.1. The Role of Learning-Related Beliefs and Motivational Dispositions

Among specific learning-related attributes, self-efficacy and self-esteem emerged as robust positive correlates of general confidence, reinforcing well-established links between self-referential beliefs and metacognition (Dapp & Roebers, 2021; Kleitman & Gibson, 2011; Roebers et al., 2012). These associations remained after the other person-level variables were controlled across multiple academic assessments. Their weaker association with resolution is consistent with a distinction between baseline self-evaluation and relative monitoring accuracy (Dapp & Roebers, 2021; Schraw, 2009). In the present concurrent data, self-efficacy and self-esteem were associated more strongly with baseline confidence than with within-person resolution; the design does not establish that these beliefs caused either pattern.

Intrinsic and extrinsic motivation were also positively associated with general confidence. Although these orientations have different implications for learning (Ryan & Deci, 2000), their coefficients showed a similar association with students’ expressed confidence. This pattern is compatible with Miele and Scholer’s (2018) metamotivational framework and extends prior evidence linking situational interest to inflated performance predictions (Senko et al., 2022). However, the concurrent design does not show that motivation raised confidence or identify the mechanism underlying the association. Motivational orientations were associated primarily with general confidence; their distinct relations with resolution require further study.

By contrast, prior knowledge was not significantly related to general confidence and was only marginally associated with resolution. This weak association may reflect the broad, static prior-knowledge measure, which may not capture the task-relevant knowledge activated during testing. Resolution is expected to depend heavily on valid, moment-to-moment cues (Dunlosky et al., 2005). Because the resolution metric summarizes within-person correspondence across assessments, it may be more closely related to context-specific cue use than to general background knowledge; this explanation remains tentative in the concurrent design.

Finally, fixed mindset was marginally associated with higher general confidence and weaker resolution. This pattern is consistent with prior evidence linking entity beliefs to overconfidence and less accurate self-assessment. For example, individuals with fixed mindsets may attend less to difficult task elements and rely more on non-diagnostic cues (Ehrlinger et al., 2016), whereas growth-oriented mindsets have been linked to more adaptive interpretations of metacognitive disfluency (Jia et al., 2025). In the present concurrent data, however, cue use was not measured; it is therefore a possible explanation rather than an observed mechanism.

By contrast, grit was not uniquely associated with metacognitive monitoring. This null finding is theoretically plausible, as prior work has linked grit more strongly to persistence and metacognitive strategy use than to the accuracy of confidence judgments themselves (Wolters & Hussain, 2015). Its shared variance with more proximal predictors, such as self-efficacy and motivation, may also have reduced its unique contribution in the full model. Similarly, test anxiety showed only a marginal negative association with general confidence and no significant relation with resolution. This weak effect may reflect the fact that trait-like anxiety is a relatively distal predictor of monitoring, whereas prior research suggests that state anxiety experienced during specific evaluative situations is more closely tied to reduced monitoring accuracy (Prinz-Weiß et al., 2023). 

Together, these findings suggest that learning-related attributes are more closely tied to between-person differences in general confidence than to differences in resolution. The weak or null unique effects for grit and test anxiety further indicate that not all adaptive learning attributes are directly associated with more accurate metacognitive monitoring. The present findings also suggest that interventions should not assume that fostering adaptive learning-related attributes will automatically strengthen resolution; rather, future work should test how supports that reduce bias in students’ confidence estimates can be integrated with supports that strengthen accurate cue use during self-evaluation.

The weaker learning attribute associations with resolution should not be taken to mean that resolution is primarily cognitive, because the measured cognitive variables also accounted for little random-slope variance. In Nelson’s (1990) monitoring framework, resolution depends on how accurately object-level performance cues are represented at the meta-level. Hierarchical accounts likewise distinguish local confidence–performance correspondence from broader, more global self-beliefs (Katyal & Fleming, 2024). On this view, stable beliefs and motivational dispositions may be associated with the baseline level of confidence, whereas the diagnostic quality and use of section-specific cues may be more proximal to resolution. This interpretation is provisional because resolution was estimated from four heterogeneous sections.

### 5.2. Implications for Theories of Intelligence and Metacognition

The overall pattern of the findings carries implications for how metacognition is situated within broader theories of intelligence. Sternberg (1984) and the APA consensus report (Neisser et al., 1996) both treat self-monitoring as integral to intelligent functioning—Sternberg through metacomponents, the executive processes that plan and evaluate performance, and Neisser et al. through capacities such as adapting to the environment and learning from experience. Yet our findings indicate that the monitoring processes that these theories place at the intellectual core are not governed by cognitive ability alone: students’ self-evaluations, when measured as general-confidence judgments, were associated more strongly with non-cognitive learning attributes than with cognitive abilities. This pattern is consistent with recent accounts suggesting that self-monitoring is not simply a direct expression of cognitive ability but may reflect mental processes that are at least partly separable from those underlying objective performance (Fleming & Daw, 2017; Fleming & Dolan, 2014; Son & Hausman, 2024). 

Viewed through a framework that distinguishes metacognitive monitoring from cognitive ability while recognizing their close interdependence, our results further suggest that different components of monitoring may relate differently to person-level characteristics. Metacognitive monitoring can be operationalized in terms of absolute accuracy (e.g., bias score) and relative accuracy (e.g., correlation gamma) (Nelson, 1990). In the present study, monitoring was operationalized as general confidence levels and resolution across tests. Stankov and colleagues characterized general confidence as a stable, domain-general disposition that is partly separable from cognitive ability and situated between cognition and personality (Stankov, 2000; Stankov & Crawford, 1997; Stankov et al., 2012)—a view supported by evidence of stable between-person confidence bias across domains (Ais et al., 2016; Mazancieux et al., 2020). Our findings extend this account by identifying self-referential beliefs and motivational orientations as person-level attributes associated with students’ general confidence levels beyond the measured cognitive variables; this incremental association does not establish independence from cognitive ability. 

## 6. Conclusions

This study primarily shows that, after accounting for actual performance, learning-related attributes—especially self-referential beliefs and motivational orientations—are associated with students’ adjusted baseline estimated performance (general confidence) across academic assessments. When added after cognitive and demographic factors, this block explained a considerable number of the individual differences in general confidence. Its associations with within-person confidence–performance correspondence (resolution) were much weaker. This pattern suggests that learning-related attributes are more closely tied to students’ baseline tendency to feel confident about their performance than to the degree of alignment between confidence and actual performance. The negative correlation between the random intercept and random slope further supports this interpretation, indicating that students with lower general confidence tended to show stronger resolution.

## 7. Limitation

Because all items were of comparable difficulty (≈50% accuracy), the grand intercept of the model captures adjusted baseline estimated performance (general confidence) across hard tasks. This does not bias the estimation of level 2 coefficients, which reflect how person-level factors predict metacognitive monitoring. However, a fixed “hard” context limits the interpretation of how individuals calibrate their confidence in relation to specific learning attributes. For instance, it remains unclear whether students with a fixed mindset become less overconfident than those with a growth mindset when tasks are easier. This moderation effect is unidentifiable in the absence of task difficulty variation. Therefore, the results should be interpreted within the context of relatively hard tasks, and generalization to tasks of varying difficulty requires further investigation.

Resolution was estimated from only four heterogeneous assessment sections. Although the likelihood-ratio test supported between-person variation in slopes, the small number of observations limits the precision of person-specific estimates and does not establish temporal stability or domain generality. Because section identity also confounds domain, format, scoring, and difficulty, observed correspondence may partly reflect contextual differences. We retained a common-slope model to estimate overall confidence–performance correspondence across these sections; section-specific effects could not be generalized without parallel sections. Thus, inferences about resolution are limited to the sections studied. Future research should include more repeated, parallel sections to examine section effects, interactions, and slope reliability.

Identified regulation was not included in the final predictor set because of its substantial empirical overlap with the retained extrinsic-motivation measure and the study’s emphasis on a parsimonious cross-construct model. Accordingly, the findings should not be generalized to identified regulation, autonomous extrinsic motivation, or comparisons among self-determination theory regulation types. Research designed specifically around self-determination theory should retain its theoretically distinct subscales and use designs and models suited to estimating their shared and unique associations with metacognitive monitoring.

The present sample was drawn from a large-scale project conducted in public primary schools in northern China, providing strong statistical power and ecological validity within this population. However, sixth-grade students represent a particular developmental stage in which metacognitive ability is still maturing, and Chinese educational and cultural contexts may shape both general confidence and the related learning attributes in ways that differ from other settings. Therefore, generalization to other age groups or educational systems should be made with caution.

Most learning-related attributes and the performance estimates were self-reported within the same wave, creating possible shared-method and response-style influences that may inflate some associations, although actual performance was assessed objectively. Moreover, although CAPDS is longitudinal, the present analyses use a cross-sectional slice of Wave 6; temporal ordering and causal direction therefore cannot be established. Replication with multi-informant or behavioral measures and longitudinal or experimental designs is needed.

## 8. Future Directions

Several directions emerge from the present findings. The divergence between general confidence and resolution suggests that future research should examine how stable person-level beliefs and motivations influence task-level judgments by distinguishing adjusted baseline differences in estimated performance from within-person responsiveness to performance fluctuations. Repeated-measures designs that capture both stable learning attributes and state-level experiences across assessments would be especially useful for clarifying how beliefs, motivations and context-specific cues jointly shape metacognitive monitoring. Experimental and intervention designs should examine the conditions under which specific beliefs and motivational orientations interact with task-level cue use. The observed gender differences also warrant further investigation, particularly regarding whether they reflect differential socialization of self-evaluative norms or cue use strategies. Finally, the weaker association between learning-related attributes and resolution suggests that educational practices aimed at fostering adaptive beliefs and motivational orientations may not be sufficient to strengthen within-person resolution. Such interventions may need to be complemented by training in task-level performance monitoring to support accurate self-evaluation.

## Figures and Tables

**Table 1:** Descriptive statistics of within-person performance and metacognitive monitoring.

	Performance Score					Judgment Score			
	Verbal Basic (1)	Reading Comprehension (2)	Math Puzzle (3)	Math Word Problem (4)		Verbal Basic (5)	Reading Comprehension (6)	Math Puzzle (7)	Math Word Problem (8)
Mean	0.57	0.51	0.50	0.40		0.78	0.74	0.73	0.68
SD	0.19	0.17	0.20	0.28		0.18	0.19	0.20	0.21
Min~Max	0.06~1	0~1	0~1	0~1		0~1	0~1	0~1	0~1
2	0.70 ***								
3	0.62 ***	0.55 ***							
4	0.57 ***	0.53 ***	0.68 ***						
5	0.15 ***	0.14 ***	0.11 ***	0.14 ***					
6	0.14 ***	0.15 ***	0.11 ***	0.15 ***		0.75 ***			
7	0.08 ***	0.07 ***	0.20 ***	0.23 ***		0.57 ***	0.57 ***		
8	0.01	0.01	0.13 ***	0.23 ***		0.51 ***	0.52 ***	0.72 ***	

^ *p* < 0.05, * *p* < 0.01, ** *p* < 0.001, *** *p* < 0.0001.

**Table 2:** Descriptive statistics of person-level variables.

	Prior Knowledge (1)	Mean Performance (2)	Self-Efficacy (3)	Self-Esteem (4)	Growth Mindset (5)	Fixed Mindset (6)	Intrinsic Motivation (7)	Identified Motivation (8)	Extrinsic Motivation (9)	Consistency of Interest (10)	Perseverance of Effort (11)	Test Anxiety (12)
Mean	73.18	0.50	3.05	15.72	3.37	2.67	1.62	3.22	2.98	2.64	3.12	2.66
SD	17.99	0.18	0.58	3.26	0.88	0.90	0.65	0.68	0.72	0.77	0.64	0.87
Min~Max	0~99.17	0.10~0.93	1~4	5~20	1~5		1~4			1~4		1~4
2	0.70 ***											
3	0.28 ***	0.22 ***										
4	0.24 ***	0.21 ***	0.60 ***									
5	−0.05 *	−0.07 ***	0.26 ***	0.23 ***								
6	−0.23 ***	−0.29 ***	−0.01	−0.04 ^	−0.07 ***							
7	−0.18 ***	−0.20 ***	−0.07 ***	−0.06 **	0.11 ***	0.30 ***						
8	0.31 ***	0.28 ***	0.60 ***	0.50 ***	0.25 ***	−0.07 ***	−0.12 ***					
9	0.25 ***	0.22 ***	0.65 ***	0.51 ***	0.25 ***	−0.03	−0.08 ***	0.79 ***				
10	0.22 ***	0.26 ***	0.23 ***	0.18 ***	−0.08 ***	−0.21 ***	−0.28 ***	0.18 ***	0.21 ***			
11	0.12 ***	0.08 ***	0.61 ***	0.61 ***	0.25 ***	−0.01 ***	−0.09 ***	0.52 ***	0.55 ***	0.18 ***		
12	−0.14 ***	−0.16 ***	−0.18 ***	−0.21 ***	0.07 ***	0.16 ***	0.18 ***	−0.08 ***	−0.15 ***	−0.38 ***	−0.14 ***	

^ *p* < 0.05, * *p* < 0.01, ** *p* < 0.001, *** *p* < 0.0001.

**Table 3:** Model comparisons across incremental linear and beta confidence model.

		Random-Intercept-Only Model	Random-Intercept–Random-Slope Baseline Model	Baseline Model + Cognitive Variables	Baseline Model + Cognitive Variables + Demographic Variables	Baseline Model + Cognitive Variables + Demographic Variables + Learning-Related Attributes
Linear Confidence Model	AIC	−12,302.99	−13,596.79	−13,772.25	−13,965.05	−14,352.51
	BIC	−12,264.66	−13,535.47	−13,680.26	−13,811.74	−14,030.56
	ΔAIC		1293.80	175.46	192.80	387.46
	ΔBIC		1270.81	144.79	131.48	218.82
	Δ*χ*^2^(df)		1301.96 (3) ***	183.46 (4) ***	208.80 (8) ***	431.46 (22) ***
Beta Confidence Model	AIC	−23,845.77	−24,664.06	−24,784.18	−25,059.66	−25,462.11
	BIC	−23,799.78	−24,595.07	−24,684.53	−24,898.68	−25,132.50
	ΔAIC		818.29	120.12	275.48	402.45
	ΔBIC		796.29	89.46	214.15	233.82
	Δ*χ*^2^(df)		911.26 (3) ***	128.12 (4) ***	291.48 (8) ***	446.46 (22) ***

^ *p* < 0.05, * *p* < 0.01, ** *p* < 0.001, *** *p* < 0.0001.

**Table 4:** Fixed effects for general confidence (random intercept).

	Linear Confidence Model			Beta Confidence Model	
	*β*	*S.E.*		*β*	*S.E.*
Grand intercept (γ_00_)	0.70 ***	0.006		0.91 ***	0.04

Cognitive variables					
between-person performance (γ_01_)	0.02 ***	0.004		0.10 ***	0.02
prior knowledge	0.005	0.004		0.03	0.02

Demographic variables					
gender	0.06 ***	0.005		0.42 ***	0.03
age	−0.001	0.002		−0.01	0.01
parental education degree	0.002	0.003		0.03	0.02
family annual income	−0.001	0.003		−0.003	0.01

Learning-related attributes					
self-efficacy	0.02 ***	0.004		0.11 ***	0.02
self-esteem	0.02 ***	0.003		0.11 ***	0.02
growth mindset	0.000	0.003		0.004	0.02
fixed mindset	0.01 ^	0.003		0.04 ^	0.02
intrinsic motivation	0.01 ^	0.003		0.05 *	0.02
extrinsic motivation	0.02 ***	0.004		0.12 ***	0.02
consistency of interest	0.001	0.003		0.01	0.02
perseverance of effort	−0.01	0.003		−0.04	0.02
test anxiety	−0.004	0.003		−0.04 ^	0.02

^ *p* < 0.05, * *p* < 0.01, ** *p* < 0.001, *** *p* < 0.0001.

**Table 5:** Fixed effects for resolution (random slope).

	Linear Confidence Model			Beta Confidence Model	
	*β*	*S.E.*		*β*	*S.E.*
Within-person performance (γ_10_)	0.04 ***	0.002		0.17 ***	0.01

Cognitive variables					
between-person performance (γ_11_)	−0.002	0.002		−0.01	0.01
prior knowledge	0.005 ^	0.002		0.02 ^	0.01

Demographic variables					
gender	−0.01 **	0.003		−0.03 *	0.01
age	−0.001	0.001		−0.004	0.01
parental education degree	0.002	0.001		0.01	0.01
family annual income	−0.001	0.001		−0.001	0.01

Learning-related attributes					
self-efficacy	−0.002	0.002		−0.01	0.01
self-esteem	0.002	0.002		0.01	0.01
growth mindset	0.000	0.001		0.001	0.01
fixed mindset	−0.004 *	0.001		−0.01 ^	0.01
intrinsic motivation	0.000	0.001		−0.004	0.01
extrinsic motivation	−0.002	0.002		−0.003	0.01
consistency of interest	0.002	0.001		0.01	0.01
perseverance of effort	−0.001	0.002		−0.01	0.01
test anxiety	0.002	0.001		0.01	0.01

^ *p* < 0.05, * *p* < 0.01, ** *p* < 0.001, *** *p* < 0.0001.

## Data Availability

The data presented in this study are available on request from the corresponding author.

## Supplementary Materials

The following supporting information can be downloaded at: https://www.mdpi.com/article/10.3390/jintelligence14080178/s1, Supplementary S1: Instruments Included in the Questionnaire; Supplementary S2: Full Model Equations Used in the Data Analysis for the Beta Confidence Model; Supplementary S3: Diagnostic Analysis of Heteroskedasticity; and Supplementary S4: Sensitivity Analysis.
