# Supplementary material for: Explaining Individual Differences in Metacognitive Monitoring: A Multilevel Analysis of Person-Level Predictors Across Academic Assessments
Source: J Intell. 2026 Aug 3;14(8):178. doi: 10.3390/jintelligence14080178 (PMC13514469; doi:10.3390/jintelligence14080178)
Supplement: Supplementary file 1 [file jintelligence-14-00178-s001.zip › jintelligence-4421007-supplementary.pdf]

Supplementary Material

Expanded Methodological and/or Technical Information

Supplementary S1. Instruments in the Questionnaire

All instruments were administered in Chinese, either originally developed in Chinese or adapted from established scales into validated Chinese versions. Reverse-worded items were recoded prior to computing composite scale scores. For transparency, the original English items, along with translated versions of self-developed or adapted scales, are provided in Supplementary S1(1)–(7).

Supplementary S1(1). Academic Assessments and Measures of Confidence Judgement

Students completed four academic assessments in two subject domains: Chinese and Mathematics. Following each assessment, they provided confidence judgments by estimating their performance on the specific test sections. The prompts were as follows:

- Chinese assessment:

  Part 1: "The first part of the Chinese assessment includes 32 knowledge items. How many correct answers do you think you provided?"

  Part 2: "The second part of the Chinese assessment includes 20 reading comprehension items. How many correct answers do you think you provided?"

- Mathematics assessment:

  Part 1: "The first part of the mathematics assessment includes 21 multiple-choice items. How many correct answers do you think you provided?"

  Part 2: "The second part of the mathematics assessment consists of problem-solving items worth a total of 16 points. How many points do you think you earned?"

Supplementary S1(2). Academic Self-Efficacy Subscale (adapted from Muris, 2001)

Items:

1. How well can you study when there are other interesting things to do?
2. How well can you study a chapter for a test?
3. How well do you succeed in finishing all your homework every day?
4. How well can you pay attention during every class?
5. How well do you succeed in passing all subjects?
6. How well do you succeed in satisfying your parents with your schoolwork?
7. How well do you succeed in passing a test?

Response options: 1 = strongly disagree, 2 = somewhat disagree, 3 = somewhat agree, 4 = strongly agree.

Supplementary S1(3). Self-Esteem Subscale (adapted from Rosenberg, 1979)

Items:

1. On the whole, I am satisfied with myself.
2. I feel that I have a number of good qualities.
3. I am able to do things as well as most other people.
4. I take a positive attitude toward myself.
5. I feel that I'm a person of worth, at least on an equal plane with others.

Response options: 1 = strongly agree, 2 = agree, 3 = disagree, 4 = strongly agree

Supplementary S1(4). Motivational Orientation Scale (adapted from Guay et al., 2010)

Intrinsic motivation

Chinese:

1. I like Chinese.
2. Chinese interests me a lot.
3. I study Chinese even when I don't have to.

Mathematics:

4. I like maths.
5. Maths interests me a lot.
6. I do mahts even when I don’t have to.

Identified regulation

Chinese:

7. I can learn many useful things by studying Chinese.
8. I choose to study Chinese to learn many things.
9. In life, it’ important to learn how to use Chinese.

Mathematics:

10. I can learn many useful things by doing maths.
11. I choose to do maths to learn many things.
12. In life, it’ important to learn how to do maths.

Controlled regulation (extrinsic motivation)

Chinese:

13. I study Chinese to get a nice reward.
14. I study Chinese to please my parents or my teacher.
15. I study Chinese to show others how good I am.

Mathematics:

16. I do maths to get a nice reward.
17. I do maths to please my parents or my teacher.
18. I do maths to show other how good I am.

Response options: 1 = always no, 2 = sometimes no, 3 = I don’t know, 4 = sometimes yes, 5 = always yes.

Supplementary S1(5). Implicit Theories of Intelligence (adapted from Dweck, 2013)

Items:

Entity view (fixed mindset)

1. You have a certain amount of intelligence, and you can't really do much to change it.
2. Your intelligence is something about you that you can't change very much.
3. To be honest, you can't really change how intelligent you are.
4. You can learn new things, but you can't really change your basic intelligence.

Incremental view (growth mindset)

5. No matter who you are, you can significantly change your intelligence level.
6. You can always substantially change how intelligent you are.
7. No matter how much intelligence you have, you can always change it quite a bit.
8. You can change even your basic intelligence level considerably.

Response options: 1 = strongly agree, 2 = disagree, 3 = somewhat agree, 4 = agree, 5 = strongly agree.

Supplementary S1(6). Grit Scale (adapted from Duckworth et al., 2007)

Items:

Consistency of Interests

1. I often set a goal but later choose to pursue a different one.
2. New ideas and new projects sometimes distract me from previous ones.
3. I become interested in new pursuits every few months.
4. My interests change from year to year.
5. I have been obsessed with a certain idea or project for a short time but later lost interest.
6. I have difficulty maintaining my focus on projects that take more than a few months to complete.

Perseverance of Effort

8. I have achieved a goal that took years of work.
9. I have overcome setbacks to conquer an important challenge.
10. I finish whatever I begin.
11. Setbacks don’t discourage me.

12. I am a hard worker.
13. I am diligent.

Response options: 1 = disagree, 2 = somewhat disagree, 3 = somewhat agree, 4 = agree

Supplementary S1(7). Test Anxiety Subscale (adapted from Pintrich, 2000)

Items:

1. I am so nervous during a test that I cannot remember facts I have learned.
2. I have an uneasy, upset feeling when I take a test.
3. I worry a great deal about tests.
4. When I take a test I think about how poorly I am doing.

Response options: 1 = disagree, 2 = somewhat disagree, 3 = somewhat agree, 4 = agree

Supplementary S2. Full Model Equations Used in the Data Analysis (Beta Confidence Model)

Level 1 (Task Level)

$$Logit(\mu) = \beta_{0i} + \beta_{1i} \cdot WithinPersonP_{ti} + \varepsilon_{ti}$$

Level 2 (Individual Level)

$$\beta_{0i} = \gamma_{00} + \gamma_{01} \cdot BetweenPersonP_i + \gamma_{02} \cdot PriorKnowledge_i$$
$$+ \sum_{p}^{P} \gamma_{0p}^{D} \cdot Demographics_{pi} + \sum_{q}^{Q} \gamma_{0q}^{T} \cdot \text{LearningAttributes}_{qi} + u_{0i}$$

$$\beta_{1i} = \gamma_{10} + \gamma_{11} \cdot BetweenPersonP_i + \gamma_{12} \cdot PriorKnowledge_i$$
$$+ \sum_{p}^{P} \gamma_{1p}^{D} \cdot Demographics_{pi} + \sum_{q}^{Q} \gamma_{1q}^{T} \cdot \text{LearningAttributes}_{qi} + u_{1i}$$

Level 3 (Class Level)

$$\gamma_{00jk} = \delta_{000k} + \upsilon_{00jk} \ ; \ \gamma_{01jk} = \delta_{010k} \ ; \ \gamma_{0njk} = \delta_{0n0k} \ ; \ \gamma_{0mjk} = \delta_{0m0k} \ ;$$

$$\gamma_{10jk} = \delta_{100k} \ ; \ \gamma_{1njk} = \delta_{1n0k} \ ; \ \gamma_{1mjk} = \delta_{1m0k}$$

Level 4 (School Level)

$$\delta_{000k} = \mu_{0000} + \upsilon_{000k} \ ; \ \delta_{010k} = \mu_{0100} \ ; \ \delta_{0n0k} = \mu_{0n00} \ ; \ \delta_{0m0k} = \mu_{0m00}$$

$$\delta_{100k} = \mu_{1000} \ ; \ \delta_{1n0k} = \mu_{1n00} \ ; \ \delta_{1m0k} = \mu_{1m00}$$

The pooled variance components of the beta confidence model on the logit scale were school = 0.01602, class = 0.03456, pupil intercept = 0.54165, pupil slope = 0.00130, and covariance = −0.00520; the reference precision was φ = 11.82 and the pupil random-effect SD on the log-precision scale was 0.945.

Supplementary S3. Diagnostic Analysis for Heteroskedasticity

We evaluated model fit and residual patterns across different model specifications. The Residuals vs. Fitted plot for the linear confidence model (Figure S1A) revealed systematic deviations. The Breusch–Pagan test, a statistical test used to detect heteroskedasticity in a regression model was also significant ($p < 0.0001$ across all 20 imputations), indicating heteroskedasticity and model misspecification. The Residuals vs. Fitted plots (Figure S1B) exhibited discernible patterns in the beta confidence model with constant dispersion, while the Breusch–Pagan test statistic was non-significant (p-values ranging from 0.09 to 0.50 across imputations). In contrast, the beta regression model with varying dispersion—allowing dispersion to vary across individuals—substantially reduced these patterns in the Pearson residuals vs. Fitted plot (Figure S1C), with Breusch–Pagan p-values ranging from 0.06 to 0.18 across imputations. Moreover, allowing variable dispersion substantially improved the goodness of fit (pooled $\Delta\chi^2(1) = 4185.26$, $p < 0.0001$). These results suggest that modeling heteroskedasticity through a varying dispersion structure is essential for achieving improved model fit.

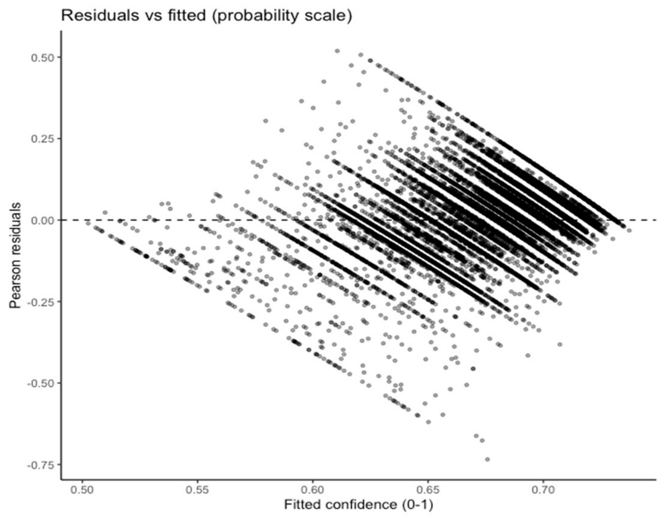

(A) Linear confidence model

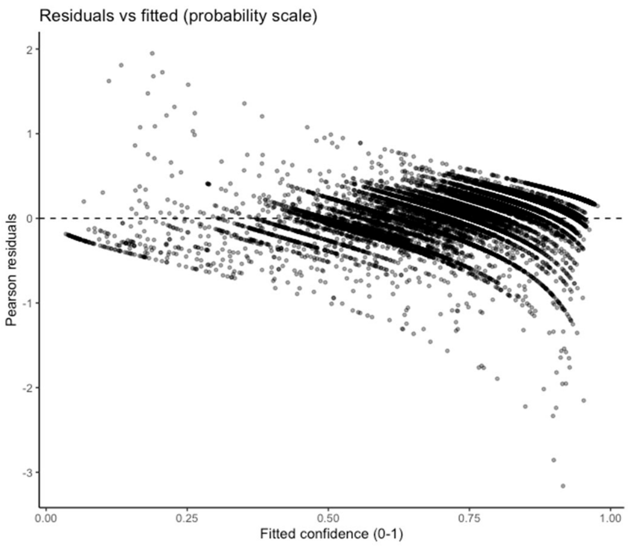

(B) Beta confidence model with constant dispersion

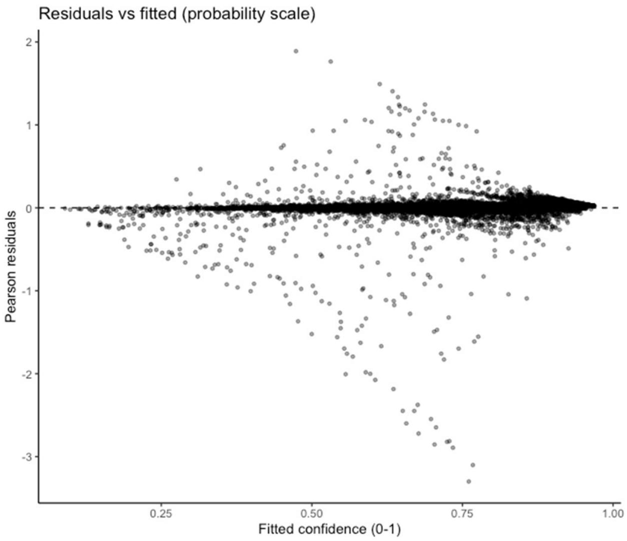

(C) Beta confidence model with varying dispersion

Figure S1 Residuals vs. Fitted plot by models

Supplementary S4. Sensitivity Analysis

As shown in the descriptive statistics of the missing percentages for variables used in the analyses (see Table S1), the increase in missingness under Criterion 2 remains within a relatively acceptable range (< 20%). Even under Criterion 2, the variable with the highest missing rate is confidence for math word problem (17.8%), while most variables remain below 15%. Assuming data are missing at random (MAR), we determined that multiple imputation was a more appropriate approach deal with missing data than listwise deletion, as dropping all cases with missing values would result in substantial data loss.

Table S1 Missing pattern by missing-data treatment

	Missing pattern	
	Criterion 1	Criterion 2
Judgment score		
verbal basic	2.46%	
reading comprehension	2.89%	
math puzzle	1.19%	
math word problem	17.83%	
Performance score		
verbal basic	2.91%	
reading comprehension	3.27%	
math puzzle	3.04%	
math word problem	3.77%	
Demographics		
gender	0.20%	
age	2.18%	
parental education degree	0.48%	
family annual income	6.31%	
Cognitive factors		
prior knowledge	1.54%	
Learning-related attributes		
self-efficacy	3.06%	12.94%
self-esteem	6.20%	6.76%
growth mindset	3.52%	14.00%
fixed mindset	4.28%	14.79%
intrinsic motivation	2.99%	4.63%
extrinsic motivation	3.01%	4.66%
consistency of interest	0.94%	0.94%

perseverance of effort	0.99%	0.99%
test anxiety	2.96%	15.30%

As detailed in the main text, we implemented three distinct estimation strategies to address missing data: (a) multiple imputation under Criterion 1, which reflects the baseline pattern of missingness; (b) multiple imputation under Criterion 2, which applies a more stringent exclusion criterion via a zero-variance filter; and (c) listwise deletion based on the same stricter exclusion criterion. To evaluate the robustness of the model results under these alternative treatments of missing data, we re-estimated the beta confidence model across all three strategies. The fixed-effect estimates exhibited a high degree of consistency across conditions, indicating that the primary inferences are not substantially affected by the choice of missing-data handling method. In contrast, estimates of the random effects—specifically the proportion reduction in variance (PRV) and correlation components—demonstrated greater variability, suggesting that these parameters are more sensitive to the underlying missing-data assumptions. These findings support the credibility of the substantive conclusions derived under the multiple imputation (Criterion 2) approach, while also highlighting the importance of considering potential instabilities in random-effect estimation when evaluating model robustness.

As shown in Table S2 & S3, across all cognitive, demographic, and learning-attribute predictors, point estimates under multiple imputation with Criterion 1 were virtually unchanged when Criterion 2 was added. Standard errors also remained stable, so most effect that reached conventional significance under Criterion 1 (e.g., self-efficacy logit$\beta = 0.13$, self-esteem logit$\beta = 0.11$, fixed mind-set logit$\beta \approx 0.04$, test anxiety logit$\beta = -0.04$, and the within-person performance slope logit$\beta = 0.17$) retained its sign and significance under

Criterion 2. Shifting to list-wise deletion under the same stricter rule produced slightly attenuated coefficients—most notably for self-efficacy (logitβ = 0.08 vs. 0.11–0.13) and self-esteem (logitβ = 0.12 vs. 0.11)—but the direction and statistical significance of every previously reliable effect remained intact. Taken together, these results show that the observed relations among confidence, performance, and learner characteristics are not an artefact of the imputation model or the exclusion threshold, supporting the credibility of the main findings.

Table S2 Fixed effects of random-intercept model by missing-data treatments

	Multiple imputation (Criterion 1)		Multiple imputation (Criterion 2)		Multiple imputation (Criterion 2)	
	β	*S.E.*	*β*	*S.E.*	*β*	*S.E.*
Grand intercept (μ_{00})	0.91 ***	0.04	0.91 ***	0.04	0.92 ***	0.04
Cognitive variables						
between-person performance (γ_{01})	0.10 ***	0.02	0.10 ***	0.02	0.15 ***	0.03
prior knowledge	0.03	0.02	0.03	0.02	0.02	0.03
Demographic variables						
gender	0.42 ***	0.03	0.42 ***	0.03	0.43 ***	0.04
age	-0.01	0.01	-0.01	0.01	0.002	0.02
parental education degree	0.03	0.02	0.03	0.02	0.03	0.02
family annual income	-0.003	0.01	-0.003	0.01	-0.002	0.02
Learning-related attributes						
self-efficacy	0.13 ***	0.02	0.42 ***	0.03	0.08 *	0.03
self-esteem	0.11 ***	0.02	-0.01	0.01	0.12 ***	0.03
growth mindset	0.01	0.02	0.03	0.02	0.02	0.02
fixed mindset	0.04 *	0.02	-0.003	0.01	0.02	0.02
intrinsic motivation	0.05 *	0.02	0.05 *	0.02	0.03	0.02
extrinsic motivation	0.10 ***	0.02	0.12 ***	0.02	0.11 ***	0.03

consistency of interest	0.02	0.02	0.01	0.02	0.03	0.02
perseverance of effort	-0.04	0.02	-0.04	0.02	-0.03	0.03
test anxiety	-0.03	0.02	-0.04 ^	0.02	-0.03	0.02

^ $p < 0.05$, * $p < 0.01$, ** $p < 0.001$, *** $p < 0.0001$

Table S3 Fixed effects of random-slope model by missing-data treatments

	Multiple imputation (Criterion 1)		Multiple imputation (Criterion 2)		Multiple imputation (Criterion 2)	
	β	*S.E.*	*β*	*S.E.*	*β*	*S.E.*
Within-person performance (γ_{10})	0.17 ***	0.01	0.17 ***	0.01	0.15 ***	0.01
Cognitive variables						
between-person performance (γ_{11})	-0.01	0.01	-0.01	0.01	-0.02	0.01
prior knowledge	0.02 ^	0.01	0.02 ^	0.01	0.03 ^	0.01
Demographic variables						
gender	-0.03 *	0.01	-0.03 *	0.01	-0.02	0.02
age	-0.01	0.01	-0.004	0.01	-0.01	0.01
parental education degree	0.01	0.01	0.01	0.01	0.01	0.01
family annual income	-0.001	0.01	-0.001	0.01	-0.01	0.01
Learning-related attributes						
self-efficacy	-0.01	0.01	-0.01	0.01	-0.01	0.01
self-esteem	0.01	0.01	0.01	0.01	0.01	0.01
growth mindset	0.002	0.01	0.001	0.01	0.003	0.01
fixed mindset	-0.02 *	0.01	-0.01 ^	0.01	-0.01	0.01
intrinsic motivation	-0.003	0.01	-0.004	0.01	-0.01	0.01
extrinsic motivation	-0.002	0.01	-0.003	0.01	-0.01	0.01
consistency of interest	0.01	0.01	0.01	0.01	0.01	0.01
perseverance of effort	-0.01	0.01	-0.01	0.01	0.01	0.01
test anxiety	0.01	0.01	0.01	0.01	0.005	0.01

^ $p < 0.05$, * $p < 0.01$, ** $p < 0.001$, *** $p < 0.0001$

Table S4 shows that the proportion reduction in variance (PRV) for the random intercept (overall confidence level) displayed a relatively stable pattern across the three missing-data treatments (23.3% and 28.5% for multiple imputation, and 26% for listwise deletion). The PRV for the random slope (resolution) also followed a similar pattern between the two imputation strategies (4.8% for Criterion 1 and 8.3% for Criterion 2). In contrast, the slope PRV diverged substantially across treatments—positive under multiple imputation but negative under listwise deletion. Likewise, the estimated intercept–slope correlation reversed direction (–0.16/–0.21 under multiple imputation vs. 0.29 under listwise deletion). Because a negative correlation is more theoretically plausible—indicating that individuals with higher overall confidence tend to be less well-calibrated—we rely on the multiple imputation results when interpreting the correlation structure. These inconsistencies underscore that random-effect estimates, particularly for the slope component, are highly sensitive to missing-data handling and should therefore be interpreted with caution.

Table S4 Random-effect estimates by missing-data treatments

	Multiple Imputation (Criterion 1)	Multiple Imputation (Criterion 2)	Listwise Deletion (Criterion 2)
PRV for random intercept			
cognitive variables	6.3%	-0.5%	3.3%
demographic variables	8.8%	8.7%	5.7%
learning attributes	16.2%	16.4%	18.8%
total variance explained	28.5%	23.3%	26.0%
PRV for random slope			
cognitive variables	-22.5%	-29.3%	-40.2%
demographic variables	19.5%	17.6%	-5.2%
learning attributes	3.5%	13.9%	-82.2%
total variance explained	4.8%	8.3%	-168.6%
Correlation			
random intercept × random slope	-0.16	-0.21	0.29
